# Effects of Consumer Dispositional Attitude on Purchase Intention in an Emerging Market

**DOI:** 10.12688/f1000research.131103.1

**Published:** 2023-04-12

**Authors:** A. Venugopal Shanbhogue, V. K. Ranjith

**Affiliations:** 1 Manipal Institute of Management, Manipal Academy of Higher Education, Manipal, Karnataka, 576104, India

**Keywords:** Consumer disposition, Consumer Ethnocentrism, Consumer Cosmopolitanism, Attitude towards Brand, Purchase Intention, Globalization, Localization, Global Brands, and Local Brands.

## Abstract

**Background:** Globalization trends have compelled multinational companies to change their marketing approach from multi-domestic marketing to global marketing. This strategy has had a major impact on the branding of companies. Due to these efforts by international companies, there has been a negative impact on local brands. Strong local brands always maintained the advantage of a high level of awareness among consumers, due to consumers’ close relationship with these brands, hence this resulted in strong and positive marketing investments in the home market. The purpose of this research is to evaluate the attitudinal dispositions towards global and local brands and purchase intention. The objective is to understand the underlying motives of consumers’ purchase decision.

**Methods**: Consumer disposition and attitude towards brands are evaluated based on respondents’ purchase intention of a select brand. Data are collected from Bangalore and Chennai using the convenience sampling method. Evaluation of the measurement model was performed using the Smart PLS 4 software.

**Results**: Findings suggest that consumer attitudinal disposition has a significant impact on consumers’ purchase intention. Attitude formation is the first step in consumers’ behavioural decision and making a purchase decision. Marketing managers/practitioners need to focus on forming this attitude in consumers’ minds, so that this attitude formation leads to a purchase decision.

**Conclusions**: This research provides theoretical and managerial implications for marketers, especially those operating in an emerging market like India as this study has examined the role of consumer disposition on attitude formation and purchase intention. From a theoretical perspective, this study developed and tested a conceptual model describing the relationship between attitudinal disposition and purchase intention.

## Introduction

Business organizations use branding to position their products in such a way that external economic and political factors do not affect their products. Branding is also evolving according to consumers’ tastes and preferences and is used as a strategic marketing tool ( Rooney, 1995). Global brands have become assets to multi-national enterprises (MNEs). Due to a significant rise in globalization, global brands carry an appeal of investing in research and development due to their ability in achieving economies of scale and scope ( Cleveland, Laroche, and Papadopoulos, 2015; Balabanis *et al*., 2019). Due to global brands’ presence all over the world, it projects certain values such as diversity and wide acceptance among consumers ( Kjeldgaard and Ostberg, 2007; Bartsch *et al*., 2016). Due to globalization, international marketers are presented with challenges as well as opportunities. Challenges arise from competitors and opportunities are from trade policy relaxation which removes the barriers of entry into another country. Consequently, consumers are faced with brands competing in the marketplace. The attitude of consumers towards global brands has been of interest to consumer behaviour researchers for decades ( Wang and Chen, 2004; Zeugner-Roth *et al*., 2015; Junaid *et al*., 2022).

Globalization trends have compelled multinational companies to change their marketing approach from multi-domestic marketing to global marketing. This strategy has had a major impact on the branding of companies. In the recent past, these international companies have focused their efforts on the development of global brands. Due to these efforts, there has been a negative impact on local brands. Most of the brands have opted to shrink their portfolio and eliminate the underperforming products in the market. Initially, this trend was seen in the fast-moving consumer goods (FMCG) sector, but recently it has occurred in other sectors as well such as banking, insurance, retail, and oil industries to name a few. Strong local brands always maintained an advantage of a high level of awareness among consumers, due to consumers’ close relationship with local brands over the years, hence this influenced strong and positive marketing investments in the home market ( Thomas, Singh, and Ambady, 2020; De Mooij, 2021, Junaid *et al*., 2022).

Product category plays a major role in consumers choosing either global or local brands. Product categories such as household products and food items may be more advantageous for local brands because of the localization bandwidth available, whereas products that are more visible goods may be appealing for global brands to invest in. Visible goods refer to the flagship products of brands; after purchasing these products, consumers feel that they are a part of global citizens and also feel that they are more cosmopolitan. These products are technically advanced and considered sophisticated to use, because these types of products are high in aspirational value, are associated with status and modernity, and are technically advanced ( Zhou and Belk, 2004; Dimofte, Johansson, and Ronkainen, 2008; Strizhakova, Coulter, and Price, 2008). One of the major advantages of a global brand is standardization. Standardization across product design, marketing, and advertising campaigns characterises a paradigm that is transferrable across countries giving global brands an edge against local competitors in a global industry ( Strizhakova *et al*., 2008; Cavusgil *et al*., 2018; Christian and Wang, 2022).

A global brand signals five underlying characteristics which form its position in the market, namely wider reach geographically and worldwide recognition; aspirational values which indicate excitement among consumers; higher perceived quality due to technical advancement and sophistication; display of ethical and environmental responsibilities; and standardization ( Holt, Quelch, and Taylor, 2004; Dimofte, Johansson, and Ronkainen, 2008; Oyserman, 2015). The characteristic of standardization is where local brands differ from global brands. Local brands adapt to local culture, traditions, and preferences, signalling their differentiation factor to consumers. Local brands also signal two more characteristics that appeal to consumers, namely uniqueness and originality. Due to their adaptation to local consumers’ preferences and tastes, local brands increase brand awareness and are also available in local markets ( Kapferer, 2005; Dimofte *et al*., 2008).

The objective of this paper is to evaluate the effects of consumer dispositional attitude on the purchase intention of global and local brands. Building on existing literature, this study aims to develop a conceptual framework, formulate hypotheses, and test the hypotheses with empirical data. The structure of the paper is as follows. The next section will cover the literature on global brands, local brands, consumer ethnocentrism, consumer cosmopolitanism, attitude towards brand, and purchase intention. This will provide a clear picture regarding consumer disposition, attitude towards brands, and purchase intention and thus provide a significant contribution to the literature on consumer behaviour.

## Literature review

Brands are widening their market reach to emerging nations, especially in a country like India. By venturing into emerging nations, managers of global brands are trying to understand consumer behaviour ( Wang, He, and Barnes, 2017; Christian and Wang, 2022). Both global and local brand firms try to localize their products to attract consumers within a specified region, while at the same time signalling their aspirations to promote global consumer culture and get global recognition; symbolic signals are an integral part of marketing the brands because it is an important part of consumer culture identity ( Manning and Uplisashvili, 2007; Manning 2010; Bardhi *et al*., 2012; Bjerrisgaard and Kjeldgaard, 2013). A brand is defined as global when it is recognized, marketed, accepted, and desired by consumers all over the world, and carries a similar product specification, marketing promotions, and positioning strategies. The products which are manufactured, advertised, and sold in a specific region or country, to meet the demand of local consumers’ tastes and needs are termed local brands ( Özsomer, 2012).

In emerging economies, there is strong competition between global and local brands, particularly due to local brands’ ability to adapt to local consumers’ needs. Due to this, local brands are gaining significant market share, and pose a stern challenge to global brands ( Dimofte *et al*., 2008). Global brands face a huge challenge to enter an emerging economy where local brands have already established a strong market presence and due to this, local brands become local icons, and use this as a marketing strategy to compete against global brands ( Steenkamp *et al*., 2003; Özsomer, 2012; Mrad and Cui, 2020). Therefore, global and local brand managers target consumers who are individualistic or cosmopolitan and collectivist or ethnocentric, respectively. Ethnocentric consumers have a collectivist attitude, where they gain information through interpersonal communication, due to which they make their purchase decision based on feelings and trust in the brand, whereas cosmopolitan consumers have individualism, acquire information through media and friends which influences their purchase decision. So, brands targeting consumers of collectivist or ethnocentric disposition make their products focus more on product features rather than making products by adding value or abstract personality traits to influence consumers who are individualistic or cosmopolitan ( De Mooij and Hofstede, 2010).

The significance of brands and brand preference has attracted researchers and practitioners in the past decade contributing to a vast body of knowledge ( Strizhakova and Coulter, 2015; Zeugner-Roth, Zabkar, and Diamantopoulos, 2015; Davvetas and Diamantopoulos, 2016; Thomas, Singh, and Ambady, 2020). The scope involved in branding research is continuously evolving and demands a thorough enquiry. Hence, to address this gap in the literature, we have considered the following consumer dispositions and their effect on consumers’ attitude towards brands and their influence on purchase intention.

### Consumer ethnocentrism

The term consumer ethnocentrism describes those consumers who believe in their own culture, see others only from their own set of values’ perspective and decline people who do not belong to their group or one who does not believe in their set of values and traditions. People with ethnocentric beliefs have an in-group and they tend to favour them and discard others who do not believe in their culture and traditions and are held in contempt ( Shimp and Sharma, 1987).

Shimp and Sharma (1987) conceptualized “consumer ethnocentrism as the beliefs held by consumers about the appropriateness, indeed the morality, of purchasing locally-made products instead of foreign-made products”. For an ethnocentric consumer, their own country’s products are superior to a foreign country’s products, even though the quality of foreign products is superior, but still, they prefer their nation’s products ( Raju, 1995; Riefler and Diamantopoulos, 2007). Ethnocentric consumers do not believe in buying foreign products as it is against their moral values, they believe it would hurt the domestic economy, thereby resulting in job losses for their fellow countrymen, not only that, but foreign brands also even pose a threat to indigenous companies ( Cleveland *et al*., 2009).

### Consumer cosmopolitanism

Cosmopolitanism dates to the eighteenth century, when people assumed themselves to be citizen of the world and their desire to adopt different cultures made them stand out from others. Cosmopolitans are those who have an intention to be recognized beyond their nation and culture and those who spend their lives seeking experiences both within and outside their nation ( Thompson and Tambyah, 1999; Cannon and Yaprak, 2002; Saran and Kalliny, 2012). In the early 1950s, many sociologists viewed cosmopolitans as outgoing people, beyond their community, and influenced by world culture more than their local traditions. Consumer cosmopolitanism is a construct that can be measured in two ways. One is that cosmopolitan consumers have cultural openness, where they are willing to adopt other cultures and are open to other cultural experiences, having a positive sense of competence towards foreign cultures.

Cosmopolitan consumers are outgoing, open to exploring other cultures and have world-mindedness. Even though they accept their local cultures, due to their yearning to be associated with a global identity they have a positive attitude towards global brands ( Cleveland *et al*., 2011). The other view is that consumer cosmopolitanism shows that a consumer embraces foreign cultures through open-mindedness and the diversity it brings due to the products available from different nations as well as cultural origins and they are positive towards consumption of foreign-made products. In this context, cosmopolitan consumers have an unprejudiced opinion towards foreign cultures and in experiencing their authenticity ( Riefler *et al*., 2012).

### Attitude towards brand

“Attitude towards brand is defined as an individual’s internal evaluation of the brand” ( Mitchell and Olson, 1981). A claim by Lutz (1991) says that for a consumer, attitudes function as a strainer for how they perceive an object. Past studies have confirmed the noteworthy effect of attitude towards purchase intention ( Zhang *et al*., 2005). Jin and Kang (2011) have established that for Chinese consumers, attitude plays an important factor that predicts purchase intention of global apparel brands.

### Purchase intention

Consumers evaluate products and services available to them before any purchase decision, so this evaluation finally results in a transaction which is termed as purchase intention ( Zeithaml, 1988). Purchase intention is referred to as the probability that consumers are going to buy the given product ( Schlosser *et al*., 2006). Alden *et al*. (1999) have argued in the literature that there is a belief that marketers are employing local and global consumption positioning for their products. Previous studies aimed to study the impact of consumers’ evaluation of global and local brands in both emerging and developed economies and their influence on purchase intention ( Alden *et al*., 2006).

**Figure 1.**
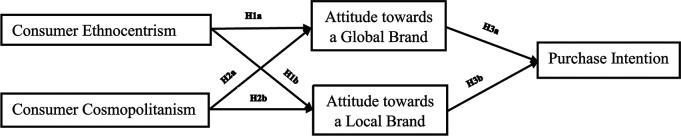
Conceptual framework - a schematic representation.

Researchers in the past have previously studied consumer dispositions such as ethnocentrism, cosmopolitanism, etc. in various research contexts and extensive research has been conducted and has had a diverse view on its impact on attitude and purchase intention. The Figure 1 below shows the conceptual framework of this study.

### Theoretical background and hypotheses development

Consumer ethnocentrism is a psycho-social construct that is relevant to an individual-level personality trait, which initially evolved from being a sociological concept ( Levine and Campbell, 1972). Consumers associated with certain beliefs, attitudes, behaviours, etc. have adopted certain social identities, which is also known as in-group, due to which the intention to buy local brand increases, hence promoting ethnocentrism and believing that they are superior to others ( Turner *et al*., 1987).

Shimp and Sharma (1987) measured consumer ethnocentrism and concluded by saying that consumers have high ethnocentric tendencies and often purchase domestic products rather than global products, and also consider domestic products to be superior, as later supported by Netemeyer *et al*.(1991) and Chryssochoidis *et al*. (2007). Consumers, when deciding to buy between local and global brands, will favour products from countries that possess a similar culture ( Sharma *et al*., 1995; Lantz and Loeb, 1996; Watson and Wright, 2000). Consumers consider the products made in their country as group related products and products of foreign countries are taken as unrelated group products, so naturally, they tend to favour products related to their group ( Verlegh, 2007).

Consumers in emerging economies are inclined to believe that local products are not as good as foreign products and favour global products more ( Balabanis *et al*., 2001; John and Brady, 2011). The consumer’s love towards their nation results in high consumer ethnocentrism ( Sharma, Shimp, and Shin, 1994). Due to this, the consumer’s evaluation of product influences in such a way that, they overestimate the domestic product’s quality and underestimate the foreign product’s quality. This would result in a positive attitude towards local products and a negative attitude towards global products ( Sharma *et al*., 1995; Watson & Wright 2000).

Hence the following hypotheses

**H1a**: There is a negative relationship between consumer ethnocentrism and attitude towards global brands.

**H1b:** There is a positive relationship between consumer ethnocentrism and attitude towards local brands.

Cosmopolitan consumers are not affected by their home culture and are open to different cultures, they transcend any boundaries or setting to experience foreign cultures and, in a way, they are motivated to achieve social status by favouring global products ( Thompson and Tambyah, 1999; Cannon and Yaprak, 2002). Cosmopolitan consumers are the ones who are outgoing, open-minded, and willing to explore other cultures with a motivation of understanding and learning from other cultures’ experiences ( Weij *et al*., 2015). Cosmopolitans from developed as well as emerging economies generally prefer buying foreign products ( Caldwell *et al*., 2006). But Riefler *et al*., (2012) argue that although cosmopolitan consumers may not prefer products from their nation, they are well-versed in their local traditions and culture.

Therefore, we hypothesise that

**H2a:** There is a positive relationship between consumer cosmopolitanism and attitude towards global brands.

**H2b:** There is a negative relationship between consumer cosmopolitanism and attitude towards local brands.

Consumers have a predisposition towards certain brands, be it global or local ( Verlegh and Steenkamp, 1999; Magnusson, Westjohn, and Zdravkovic, 2011). This may be either positive or negative, and either way, it influences their purchase decision. Consumers’ attitude towards brands originates from their past experiences, and it affects their purchase intention ( Costa *et al*., 2016; Herz and Diamantopoulos, 2017).

Hence the following hypotheses

**H3a:** There is a significant relationship between attitude towards global brands and purchase intention.

**H3b:** There is a significant relationship between attitude towards local brands and purchase intention.

## Methods

This study is aimed at understanding the underlying motives of consumer preference towards global and local brands. This research is based on a positivist paradigm, deductive reasoning, and quantitative approach. Aligned with this objective, this research adopts a quantitative approach built on an empirical survey to evaluate the conceptual model. Survey-based research allows us to understand the relationship between consumer ethnocentrism, consumer cosmopolitanism, attitude towards brand, and purchase intention. The target respondents were chosen from the metropolitan cities of Bangalore and Chennai in the southern part of India, the respondents were between the age group of 18 to 60 years. These two cities were selected for this study because, historically, states with higher gross added value (GVA) shares, including Maharashtra, Madhya Pradesh, Punjab, and Haryana, had declines whereas states like Tamil Nadu and Karnataka experienced increases over the past ten years (Kpmg, 2021).

Based on a conceptual model, a questionnaire was developed in the English language. 800 questionnaires were distributed during data collection in the metropolitan cities of Bengaluru and Chennai. The sample observations collected in this study had either missing values or incomplete surveys in 44 survey forms. These 44 forms were eliminated from the data analysis, and finally, 756 responses were considered for the study. The responses of all the 756 respondents were separately investigated to verify the probability of the presence of straight lining. No straight-lining response pattern was found in any of the sample cases. Hence, there existed no necessity to remove any observations of any particular case.

### Measures

The questionnaire consisted of scales concerning constructs of consumer ethnocentrism with 5 measurement items, consumer cosmopolitanism with 12 measurement items, attitude towards brands with 5 measurement items, and purchase intention with 5 measurement items. The study used a 5-point Likert scale labelled from 1 (strongly disagree) to 5 (strongly agree). For the measurement of the independent variables and dependent variables. Table 1 has a list of the measuring items.

**Table 1.** Research construct and items included in the questionnaire.

Sl.No	Statement	Source
1	Consumer Ethnocentrism (CET)	( Verlegh, 2007)
	CET Item 1: Indian people should not buy foreign products, this hurts Indian businesses and causes unemployment	
	CET Item 2: It is not right to purchase foreign products because this puts Indians out of jobs	
	CET Item 3: A real Indian should always buy Indian products	
	CET Item 4: I always prefer Indian products over foreign ones	
	CET Item 5: We should purchase products manufactured in India, instead of letting other countries get rich off us	
2	Consumer Cosmopolitanism (COS)	( Riefler *et al*., 2012)
	COS Item 1: When traveling, I make a conscious effort to get in touch with the local culture and traditions	
	COS Item 2: I like having an opportunity to meet people from many different countries	
	COS Item 3: I like to have contact with people from different cultures	
	COS Item 4: I have got a real interest in other countries	
	COS Item 5: Having access to products coming from many different countries is valuable to me	
	COS Item 6: The availability of foreign products in the Indian market provides valuable diversity	
	COS Item 7: I enjoy being offered a wide range of products coming from various countries	
	COS Item 8: Always buying the same local Indian products becomes boring over time	
	COS Item 9: I like watching movies from different countries	
	COS Item 10: I like listening to music of other cultures	
	COS Item 11: I like trying original dishes from other countries	
	COS Item 12: I like trying out things that are consumed elsewhere in the world	
3	Attitude towards a Brand (ATB)	( Spears and Singh, 2004)
	ATB Item 1: This brand is unappealing/appealing	
	ATB Item 2: This brand is bad/good	
	ATB Item 3: This brand is unpleasant/pleasant	
	ATB Item 4: This brand is unfavourable/favourable	
	ATB Item 5: This brand is unlikable/likable	
4	Purchase Intention (PI)	( Spears and Singh, 2004)
	PI Item 1: I will never/definitely buy this brand	
	PI Item 2: I definitely do not intend to buy/definitely intend to buy this brand	
	PI Item 3: I have very low/high purchase interest	
	PI Item 4: I would definitely not buy/definitely buy it	
	PI Item 5: I would probably not/probably buy it	

### Sampling design and data collection

Sampling is done in two stages. In the first stage, the sampling method is employed to draw the products for the consumer survey. Therefore, the judgment sampling technique is employed under the nonprobability sampling method to draw products and brands from the FMCG sector. The FMCG sector is identified for the study using these three criteria: (i) availability of global and local brands in the same product category, (ii) low price point of the products, and (iii) accessibility to a broad range of consumers ( Narang, 2016).

The product category with higher penetration and higher market share identified for this research is biscuits from food and beverages. Since India is the highest consumer of biscuits in the world and these products have a low price point, easily accessible to consumers, it has consumers from all the strata (TRA Research, 2022).

In the second stage of sampling, data were collected using the convenience sampling method. Convenience sampling was particularly relevant in this research because researchers needed to choose respondents who are already familiar with the chosen brands ( Narang, 2016). In addition to this, researchers chose respondents who were reasonably knowledgeable about global and local brands. A pilot study was done with a sample size of 84 to ensure that there are no major flaws in the study instrument and to check the reliability and validity of the scale used in this research.

### Data analysis

The demographic description of the data was conducted using IBM-SPSS software (RRID:SCR_019096), and the evaluation of the measurement model was done using Smart PLS 4 (RRID:SCR_022040). Investigating the convergent validity and discriminant validity of the measurement model allowed for an evaluation of its quality. Outer loadings were checked to negate the measurement items with poor loadings, and such items were deleted from the analysis ( Gefen and Straub, 2005).

### Ethical considerations

Before distributing the survey questionnaire, all survey participants provided their written informed consent. Before asking for the respondents' signed informed consent, an information sheet outlining the research's goals, findings, and consequences was included in the form's content. It contains statements stating that any personal information will be kept confidential and that the data obtained will be used only for research and publication purposes (see Extended data, Shanbhogue & Ranjith, 2023). In addition, the Manipal Institute of Management ethics committee and doctoral advisory committee panel gave their approval before conducting this research (date of approval 24.02.2022).

## Results

Descriptive statistics were estimated, and the output is presented in Table 2. The full data set can be found in the *underlying data* ( Shanbhogue & Ranjith, 2023).

**Table 2.** Social-demographic information.

Sl. No.	Demographic Variables	Frequency	Percentage
1	Age in years		
	20 to 29	321	42.5
	30 to 39	2	0.3
	40 to 49	231	30.6
	50 to 59	202	26.7
	Total	756	100
2	Gender		
	Male	390	51.6
	Female	366	48.4
	Total	756	100
3	Educational Qualification		
	Graduate	233	30.8
	Postgraduate	356	47.1
	Other	167	22.1
	Total	756	100
4	Current Relationship Status		
	Married	397	52.5
	Single, Never Married	359	47.5
	Total	756	100
5	Current Employment Status		
	Self Employed	160	21.2
	Employed	537	71
	Other	59	7.8
	Total	756	100
6	Household income per month		
	Rs 20000 to 40000	223	29.5
	Rs 41000 to 60000	373	49.3
	Rs 61000 to 80000	160	21.2
	Total	756	100

Age-wise distribution of the sampled respondents shows that 42.5 percent of the respondents belong to the age group of 20-29 years, 0.3 percent of the respondents belong to the age group of 30-39 years, 30.6 percent belong to the age group of 40-49 years, and 26.7 percent of the respondents belong to the age group of 50-59 years. The sample population comprised 51.6 percent of males, and 48.4 percent of females.

Among the educational qualification category, 30.8 percent of the respondents are graduates, 47.1 percent of the respondents are postgraduates, and 22.1 percent of the respondents belong to others. In terms of relationship status, 52.5 percent of the respondents are married, and 47.5 percent of the respondents are single, never married. The employment status shows that 21.2 percent of the respondents are self-employed, 71 percent of the respondents are employed, and 7.8 percent of the respondents belong to others. 29.5 percent of the respondents have a household income of Rs 21000 to 40000 per month, 49.3 percent of the respondents earn Rs 41000 to 60000 monthly income, and 21.2 percent of the respondents have a monthly income of Rs 61000 to 80000.

### Reliability assessment

Hair *et al*. (2012) describe that reliability and validity need to be assessed for the measurement model. The specific measures for internal consistency and reliability are composite reliability, convergent validity, and discriminant validity. As per Hair *et al*. (2012), composite reliability is used to evaluate internal consistency, and average variance extracted (AVE) is used to determine convergent validity. The researchers adopt the Fornell-Larcker criterion and the heterotrait-monotrait ratio (HTMT) to assess the discriminant validity Hair *et al*. (2012).

The first criterion of composite reliability has been assessed for internal consistency. Hair *et al*. (2012) recommend composite reliability for measuring the internal consistent reliability of the PLS-SEM approach rather than Cronbach’s alpha value. The threshold value for composite reliability is above 0.60. It is evident from Table 3, that internal consistent reliability among all constructs of the measurement model is acceptable as the values are above the threshold values. Since Cronbach alpha value and composite reliability value for all constructs are above 0.70, it is declared that the constructs used for the study have internal consistent reliability Hair *et al*. (2012). Therefore, all the values of composite reliability are greater than the average variance extracted for all the constructs. The average variance extracted (AVE) for each of the constructs exceeds the threshold value of 0.50, thus confirming the convergent validity of the scale ( Nunnally, 1994).

**Table 3.** Reliability assessment.

Variables	Indicators	Cronbach's Alpha	Composite reliability	Average variance extracted
Attitude towards global brand	ATBG	0.954	0.955	0.956
Attitude towards local brand	ATBL	0.912	0.914	0.919
Consumer Ethnocentrism	CET	0.917	0.931	0.923
Consumer Cosmopolitanism	COS	0.798	0.823	0.830
Purchase Intention (Global brand)	PIG	0.941	0.952	0.944
Purchase Intention (Local brand)	PIL	0.945	0.957	0.948

### Discriminant validity

Discriminant validity is about measuring the individuality of a construct among other constructs of the measurement model. It measures the extent of the distinctness of one construct from the other constructs. The researchers use the Fornell-Larcker criteria, and the HTMT to assess the discriminant validity.

### Fornell-Larcker criteria

The results of the Fornell-Larcker criteria for the discriminant validity in Table 4 demonstrate the square root of every construct exceeding its correlations with the other construct. Therefore, it is evident from the result that discriminant validity exists among the constructs used for the study.

**Table 4.** Fornell-Larcker criteria.

	ATBG	ATBL	CET	COS	PIG	PIL
ATBG	0.978					
ATBL	0.101	0.959				
CET	-0.022	-0.548	0.961			
COS	-0.108	0.002	-0.187	0.911		
PIG	0.802	0.201	-0.062	-0.100	0.972	
PIL	0.114	0.784	-0.507	-0.264	0.252	0.973

ATBG- Attitude towards global brands; ATBL- Attitude towards local brands; CET- Consumer ethnocentrism; COS- Consumer cosmopolitanism; PIG- Purchase intention of global brands; PIL- Purchase intention of local brands.

### HTMT

Henseler *et al*. (2015) proved through their simulation models that the HTMT ratio is better than the Fornell-Larcker criterion for discriminant validity. The heterotrait-hetro method correlations geometric mean is divided by monotrait-hetro method correlations average to derive the HTMT ratio.

As per Henseler *et al*.’s (2015) suggestion, to understand the discriminant validity that has been established among the constructs, the HTMT ratio value should be below 0.90. Table 5 indicates a good discriminant validity among the constructs since the HTMT ratio of all the constructs among other constructs is less than 0.80. After assessing the reliability, construct validity, and discriminant validity researchers find that there is good reliability and validity among the indicators. Therefore, structural model assessment and hypotheses testing are done in the next section.

**Table 5.** Heterotrait-monotrait ratio (HTMT).

	ATBG	ATBL	CET	COS	PIG	PIL
ATBG						
ATBL	0.108					
CET	0.024	0.596				
COS	0.122	0.064	0.219			
PIG	0.843	0.216	0.065	0.112		
PIL	0.12	0.840	0.538	0.304	0.265	

### Structural model and hypotheses testing

**Figure 2.**
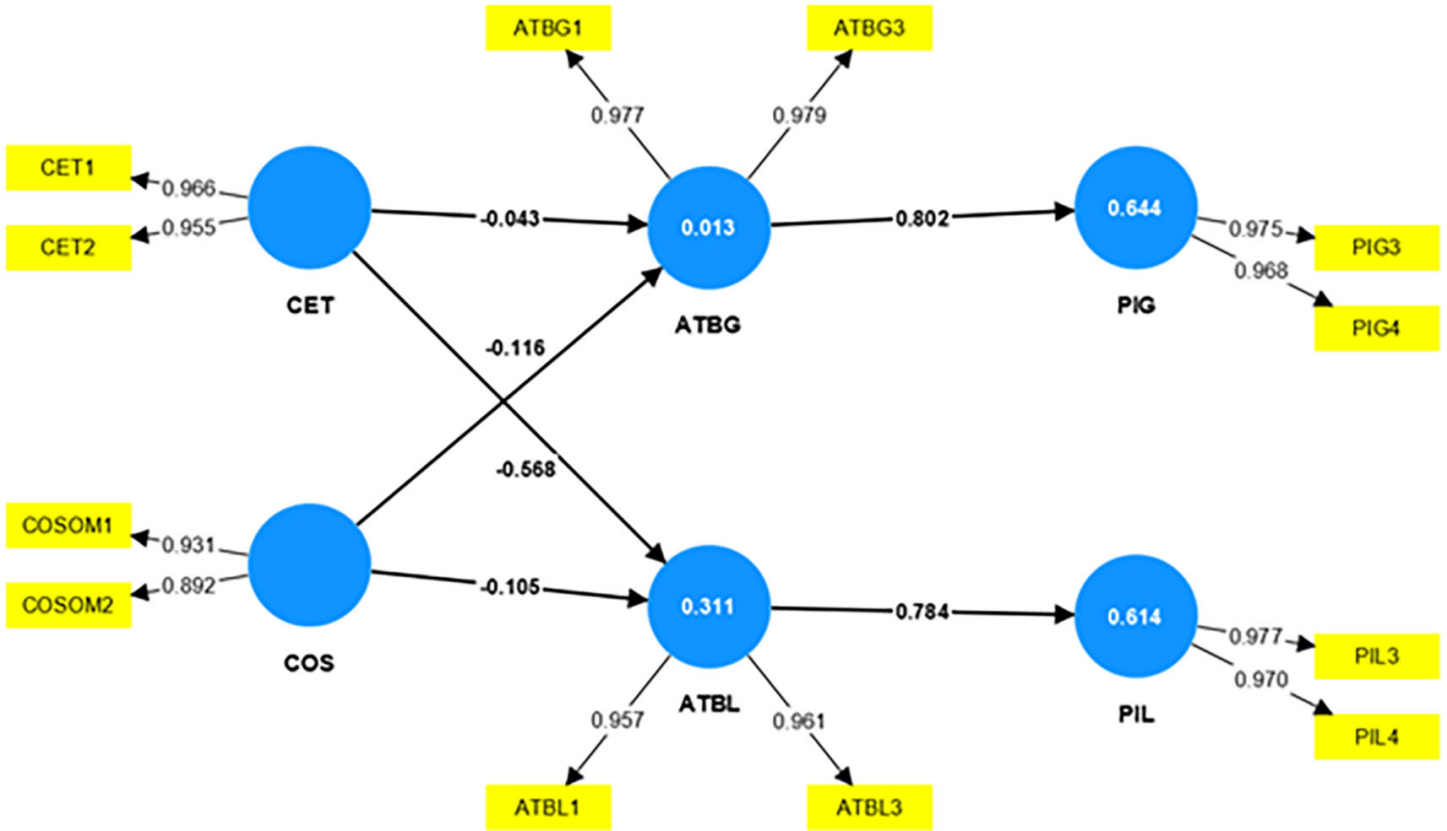
Structural model assessment.

The researchers validate the model with the PLS-SEM approach and examine the conceptual framework developed based on the literature support. The researchers adopt the PLS-SEM algorithm to compute the path coefficients of the structural model. The Figure 2 shows the structural model assessment of this study.

A strong relationship between the constructs exists if the path coefficient values are not near zero. If the values are near zero, it indicates a weak relationship between the constructs Hair *et al*. (2012). The bootstrapping technique evaluates the significance level of the relationship between the constructs. The evaluation of the structural model will show the significance level, and the t-value above 1.96 declares the relationship between the constructs is significant.

Since the coefficient determination is the criterion by which the structural model is measured for its predictive accuracy ( Henseler *et al*., 2012; Hair *et al*., 2012), the coefficient of determination, i.e., the R ^2^ value is considered to validate the model. The model stands weak, with a value of R ^2^, the coefficient of determination being 0.013 for attitude towards global brands, weak with a value of 0.311 for attitude towards local brands, reasonably strong with a value of 0.644 for purchase intention of global brands, and 0.614 for purchase intention of local brands as shown in Table 6.

**Table 6.** R ^2^ Values.

	R ^2^	R ^2^ Adjusted
ATBG	0.013	0.008
ATBL	0.311	0.308
PIG	0.644	0.643
PIL	0.614	0.613

### Model fit

For model fit, the value of Standardized Root Mean Square Residual (SRMR) should be less than the threshold value of 0.08 or 0.10 according to Henseler *et al*. (2014). Since the value of SRMR is less than the threshold value as shown in Table 7, it represents a good model fit. Another indicator of a good model fit is Normed Fit Index (NFI). According to Lohmöller (1989), the value of NFI lies between 0 and 1, the closer the NFI is to 1, the better the fit. Since the value of NFI is closer to 1 as shown in Table 7, it is considered a good model fit.

**Table 7.** Model fit.

	Saturated model	Estimated model
SRMR	0.044	0.099
Chi-Square	1053.52	1188.913
NFI	0.763	0.733

The structural model assessment and bootstrapping results in Table 8 summarize the path coefficient values, t-values, p-values, significance levels, and bootstrap confidence intervals for the model. The results of hypothesis testing for the model are illustrated in Table 9.

**Table 8.** Path coefficients, t values, p values, and Significance level.

	Path coefficients	t values	p values	Significance level
CET -> ATBG	-0.043	0.661	0.509	+
CET -> ATBL	-0.568	11.787	0.000	***
COS -> ATBG	-0.116	1.607	0.108	+
COS -> ATBL	-0.105	1.595	0.111	+
ATBG -> PIG	0.802	26.655	0.000	***
ATBL -> PIL	0.784	19.533	0.000	***

^**+**^ p < 0.10.

* p < 0.05.

** p < 0.01.

*** p < 0.001.

**Table 9.** Hypothesis outcomes.

Hypothesis No.	Hypothesis statement	Results
H1a	There is a negative relationship between consumer ethnocentrism and attitude towards global brands.	Not supported
H1b	There is a positive relationship between consumer ethnocentrism and attitude towards local brands.	Supported
H2a	There is a positive relationship between consumer cosmopolitanism and attitude towards global brands.	Not supported
H2b	There is a negative relationship between consumer cosmopolitanism and attitude towards local brands.	Not supported
H3a	There is a significant relationship between attitude towards global brands and purchase intention.	Supported
H3b	There is a significant relationship between attitude towards local brands and purchase intention.	Supported

## Discussion

This study examined the effects of consumer disposition on consumers’ attitude towards a brand and their influence on purchase intention. It provides a better understanding of the underlying motives of consumers in their purchase decision. It shows how consumers form attitudinal dispositions towards brands. In this study, consumer ethnocentrism forms a negative attitude toward global brands (H1a) and forms a positive attitude toward local brands (H1b), these findings are compatible with other studies ( Özsomer, 2012; Strizhakova and Coulter, 2015; Thomas, Singh, and Ambady, 2020). Ethnocentric consumers do favour local brands in comparison with global brands. The sense of supporting the local economy, and local culture is stronger compared to showing a favourable attitude in purchasing global brands.

Consumer cosmopolitanism is not influencing consumers in forming a favourable attitude towards global brands (H2a), this finding is not in accordance with previous studies ( Riefler *et al*., 2012; Weij *et al*., 2015; Mrad and Cui, 2020). The inference from this finding could be that consumers do not necessarily prefer purchasing global brands in the low involvement category where an alternative to that particular brand is offered by a local brand. Consumer cosmopolitanism does not show a negative influence on consumers buying local brands (H2b), this finding does not concur with previous studies ( Zeugner-Roth *et al*., 2015; Mrad and Cui, 2020). This shows that cosmopolitan consumers may prefer buying global brands over local brands which shows their cultural openness, and open-mindedness but they may also not outrightly reject local brands originating from their own country or culture.

Consumer ethnocentrism and consumer cosmopolitanism both form a strong attitude in consumers towards global and local brands (H3a) and (H3b). This finding supports past research ( Xie *et al*., 2015; Eren-Erdoğmuş and Dirsehan, 2017; Cavusgil *et al*., 2018). Consumer attitudinal disposition has a significant impact on consumers’ purchase intention. Attitude formation is the first step in consumers’ behavioural decision and making a purchase decision. Marketing managers need to focus on forming this attitude in consumers’ minds so that this attitude formation leads to purchase decision. If the consumer is ethnocentric then brands should focus on integrating local culture, and traditions in their products so that it leads to positive attitude formation and ultimately translates into purchase transactions, similarly, if the consumer is cosmopolitan then brands should signal global attributes such as wider global reach, wider global acceptance of their products, and global consumer culture.

## Conclusion

The theoretical and managerial implications of this study are significant for marketers, especially those working in an emerging market like India as this study has examined the role of consumer disposition on attitude formation and purchase intention. From a theoretical perspective, this study developed and tested a conceptual model based on attitudinal disposition and purchase intention. It suggests that ethnocentric consumers have a strong sense of oneness among their in-group, and significantly support local culture, traditions, and economy which leads to higher purchase intention towards local brands, and they form a negative attitude towards global brands which leads to lower purchase intention. It is also noted from the findings that cosmopolitan consumers do not have a negative attitude towards local brands which implies that if there is an alternative to the global brand in the market, cosmopolitan consumers will not outrightly reject the local brand in favour of the global brands.

From managerial contribution, this study can help managers understand the phenomenon of positive attitude formation which in turn leads to purchase intention. The strategies could be developed in accordance with these findings to leverage the target consumer groups. Consequently, these research findings are valuable from more than just an academic standpoint and also for managers/practitioners of brands in an emerging economy like India to strategically plan marketing campaigns, promotions, and positioning of their brands. From a managerial implications perspective, it can be concluded that managers need to adopt a flexible strategy to excite the consumers to buy their products and at the same time make their brand more appealing not only at home but also in foreign markets ( Zeugner-Roth *et al*., 2015).

### Limitations and scope for further research

Firstly, the sample, given its homogeneity, does not perfectly represent the entire spectrum of consumers, as a high number of respondents in this study are college/university students. The difference among educational qualifications still exists, which might lead to differences in their behavioural aspects of attitude formation or attitudinal dispositions. Secondly, this study discusses a specific brand or product category, hence this limitation is also one of the scopes for future research to include more product categories or a set of specific brands for the consumers to evaluate and compare before making a purchase decision. Finally, this study has not considered any moderators in the research model. Top researchers in this discipline have advocated that consumer disposition constitutes differently depending upon the contextual factors in terms of consumption, therefore future research should replicate this study by accounting for such aforementioned factors influencing consumers’ attitude and purchase intention.

## Data Availability

Figshare: Data File.xlsx. https://doi.org/10.6084/m9.figshare.21988643 ( Shanbhogue & Ranjith, 2023). This project contains the following underlying data:
• Data File.xlsx This project contains the following extended data:
• Questionnaire Parle G.docx (the survey form used during data collection) • Questionnaire Unibic.docx (the survey form used during data collection) Data are available under the terms of the Creative Commons Zero “No rights reserved” data waiver (CC0 1.0 Public domain dedication).
