# Supplementary material for: Effects of Consumer Dispositional Attitude on Purchase Intention in an Emerging Market
Source: F1000Res. 2024 Feb 28;12:384. Originally published 2023 Apr 12. [Version 3] doi: 10.12688/f1000research.131103.3 (PMC10904944; doi:10.12688/f1000research.131103.3)
Supplement: Peer review history

# Reviewer report: Reviewer response for version 2

**DOI:** 10.5256/f1000research.159017.r235138

**Published:** 2024-02-15

**Authors:** Kamelia Chaichi

**Affiliations:** 1 Queen Margaret University, Musselburgh, Scotland, UK

**Summary:** The article investigates the impact of globalization on consumer attitudes towards global and local brands in the context of Bangalore and Chennai. With globalization, multinational companies have shifted towards global marketing strategies, affecting the visibility and perception of local brands negatively. The research aims to understand consumer attitudes towards both types of brands and their purchase intentions. Data was collected through convenience sampling in Bangalore and Chennai, and the measurement model was evaluated using Smart PLS 4 software.

**Introduction was very good:** the study seeks to explore consumers' attitudes towards global and local brands, focusing on the Indian markets of Bangalore and Chennai. Understanding consumer motivations and attitudes is crucial for marketers to devise effective strategies amidst globalization trends.

**Methods:** The research employed convenience sampling to gather data from consumers in Bangalore and Chennai. The Smart PLS 4 software was utilized to assess the measurement model and analyze the relationships between variables. While convenience sampling offers practical advantages, its limitations in representing diverse consumer segments should be acknowledged.

**Results:** was explained clearly

**Implications:** The study contributes theoretical insights into consumer behavior in emerging markets like India. However, the article could enhance its contribution by delving deeper into the implications of its findings, particularly in addressing the modest R2 values. Justification and further exploration of the weak R2 values would enhance the robustness of the research findings. Additionally, practical implications derived from the study could be expanded to offer actionable insights for marketers navigating the global-local brand landscape.

Is the work clearly and accurately presented and does it cite the current literature?

Yes

If applicable, is the statistical analysis and its interpretation appropriate?

Partly

Are all the source data underlying the results available to ensure full reproducibility?

Yes

Is the study design appropriate and is the work technically sound?

Yes

Are the conclusions drawn adequately supported by the results?

Yes

Are sufficient details of methods and analysis provided to allow replication by others?

Yes

Reviewer Expertise:

Management, Marketing, Branding, human resource

I confirm that I have read this submission and believe that I have an appropriate level of expertise to confirm that it is of an acceptable scientific standard, however I have significant reservations, as outlined above.

# Reviewer report: Reviewer response for version 2

**DOI:** 10.5256/f1000research.159017.r224402

**Published:** 2024-02-14

**Authors:** Dr Irfan Sabir

**Affiliations:** 1 Faculty of Management Studies, University of Central Punjab, Lahore, Pakistan

The author still need to add latest references related to the variables (Consumer ethnocentrism, Consumer cosmopolitanism, Attitude towards brand, Purchase intention), and, Theoretical background and hypotheses development.

Is the work clearly and accurately presented and does it cite the current literature?

No

If applicable, is the statistical analysis and its interpretation appropriate?

Yes

Are all the source data underlying the results available to ensure full reproducibility?

Yes

Is the study design appropriate and is the work technically sound?

Partly

Are the conclusions drawn adequately supported by the results?

Partly

Are sufficient details of methods and analysis provided to allow replication by others?

Yes

Reviewer Expertise:

Consumer Behavior, New Product Development, Social Entrepreneurship

I confirm that I have read this submission and believe that I have an appropriate level of expertise to confirm that it is of an acceptable scientific standard, however I have significant reservations, as outlined above.

# Reviewer report: Reviewer response for version 2

**DOI:** 10.5256/f1000research.159017.r224403

**Published:** 2023-11-21

**Authors:** Savita Bhat

**Affiliations:** 1 School of Humanities, Social Sciences and Management, National Institute of Technology Karnataka, Surathkal, Karnataka, India

There are no further comments.

Is the work clearly and accurately presented and does it cite the current literature?

Yes

If applicable, is the statistical analysis and its interpretation appropriate?

Partly

Are all the source data underlying the results available to ensure full reproducibility?

Yes

Is the study design appropriate and is the work technically sound?

Yes

Are the conclusions drawn adequately supported by the results?

Partly

Are sufficient details of methods and analysis provided to allow replication by others?

Yes

Reviewer Expertise:

Emerging economies; Firm competitiveness; quantitative techniques in economics and management

I confirm that I have read this submission and believe that I have an appropriate level of expertise to confirm that it is of an acceptable scientific standard.

# Reviewer report: Reviewer response for version 1

**DOI:** 10.5256/f1000research.143913.r185052

**Published:** 2023-07-19

**Authors:** Dr Irfan Sabir

**Affiliations:** 1 Faculty of Management Studies, University of Central Punjab, Lahore, Pakistan

1. Research problem is not justified, i.e. why was this study required to be conducted, and why in India?

 2. Majority of references are older than 5 years. Need to cite latest references, i.e. within past 3 years.

 3. Literature is well written.

 4. Consumer ethnocentrism has been discussed as a psycho-social construct, yet, the theoretical underpinnings is missing, i.e. which theory supports the conceptual model of this research.

 5. Sampling design is not appropriate, as the premise of this study is "Global vs Local Brands", so there seems to be a need for respondents who belong to more diverse cultures / backgrounds, but the respondents for this study are only from two cities in India. Either responses from some other country should be added or more justification for current respondents be provided.

 6. Product category selected (Biscuits) does not seem to be appropriate, as being "low involvement products". More justification be added.

 7. Analysis done is appropriate.

Is the work clearly and accurately presented and does it cite the current literature?

No

If applicable, is the statistical analysis and its interpretation appropriate?

Yes

Are all the source data underlying the results available to ensure full reproducibility?

Yes

Is the study design appropriate and is the work technically sound?

Partly

Are the conclusions drawn adequately supported by the results?

Partly

Are sufficient details of methods and analysis provided to allow replication by others?

Yes

Reviewer Expertise:

Consumer Behavior, New Product Development, Social Entrepreneurship

I confirm that I have read this submission and believe that I have an appropriate level of expertise to confirm that it is of an acceptable scientific standard, however I have significant reservations, as outlined above.

# Response

**Published:** 2023-07-31

**Authors:** Venugopal Shanbhogue

**Affiliations:** Manipal Institute of Management, India

**Response to Query no 1:**

 In an emerging economy like India, consumers with expendable income have more choice in terms of global brands and local brands. India is an emerging economy and with high ethnocentric tendencies. So, the need for study is to analyze the effects of consumer disposition and attitude towards brands on purchase intention. Therefore, the research problem states that “Does consumer disposition towards global and local brands have an impact on attitude towards brand and their purchase intention?”

 Consumers in emerging market have a negative attitude towards global brands and globalization because of the fear that it will eradicate their local culture due to the burden of western values by capitalistic global brands (Martin and Rey, 2006). However, consumers may not have intention to purchase due to multiple dispositions such as consumer ethnocentrism, consumer cosmopolitanism, nationalism (Fernandez-Ferrin *et al*., 2015; Bartikowski and Walsh, 2015). Marketing researchers over the period have recognized cultural identity markers which received a lot of attention due to nationalistic views worldwide. In the marketing literature, national identity is investigated and further classified into a belief system and ethnocentric tendencies (Varman and Belk, 2009; Westjohn et al., 2012; Prince et al., 2020).

 Although studies on constructs such as consumer ethnocentrism, consumer cosmopolitanism, attitude towards brands, and purchase intention were carried out previously in branding literature (Zeugner-Roth *et al*., 2015; Thomas *et al*., 2020). Limited attempts have been made to understand the combining effect of positive and negative disposition towards global and local brands (Srivastava *et al*., 2020). This happens when consumers embrace global consumer culture by purchasing or experiencing global products and services yet find themselves attached to their own local culture and traditions and cannot avoid being influenced by them. And consumers who are oriented more towards local consumer culture are aware of global brands and the threat it poses to indigenous brands; they are more grounded in nationalistic and patriotic behavior in an emerging economy like India (Oyserman, 2015, Mandler *et al*., 2021).

 There has been a considerable amount of research carried out in branding, specifically concerning global and local brands. Since the findings from past literature indicate contradictory results regarding brand preference among consumers (Eren-Erdogmus and Dirsehan, 2017; Cavusgil *et al*., 2018). Citing these contradictions (Balabanis *et al*., 2019) have recently called for new avenues of research in assessing consumer preferences for global brands over local brands or vice versa in emerging markets like India.

 In this study we aim to address this gap in knowledge and understand the underlying motives of consumer preferences and the factors influencing consumers' purchase intention of global and local brands.

**Response to Query no 2:**

 Authors have included recent references i.e. within three years in this research, however the context of study is such that paucity of research in this area has compelled us to cite majority of the references from the time period when the research activity in this area of study was at its peak.

**Response to Query no 4:** 

 Consumer behaviour in emerging economies affects how consumers purchase brands, how markets are segmented, and how trade is conducted internationally. These opinions of foreign nations and their products can be positive or negative (Riefler, 2017; Papadopoulos et al., 2018). Consumer ethnocentrism, which can be seen as an exclusive response to the inflow of both local and foreign cultures, is one example of a disparaging nationalistic trait (Riefler, 2017). Entry strategies like partnerships with local partners will be chosen based on the market knowledge of customer ethnocentric tendencies (Paul, 2019).

 By offering a research model that incorporates numerous exclusionary responses and in-group and out-group orientations that are ignored in the marketing literature (Zeugner-Roth et al., 2015), the current study advances earlier research (Riefler and Diamantopoulos, 2007; Bartikowski and Walsh, 2015) and benefits from social identity theory. The theory of social identity is the foundation of the current investigation (Tajfel and Turner, 1979). In-group favouritism, which is defined as favouring members of one's group (in-group) above members of other groups, is examined by social identity theory (Turner et al., 1987), and it is helpful to understand the connections between consumer ethnocentrism and aversion to buying foreign goods. It also shows that individuals are driven to see their in-group as advantageous and superior to out-groups (Li et al., 2015).

**Response to Query no 5:**

**Study Context**

 India is an appropriate context to study global brand purchase phenomena. The country has witnessed significant changes in living standards because of an upsurge in income levels, creating opportunities for both domestic and multinational companies. These evolving trends inferred auspicious financial possibilities for global and local brands. The target population for this study consists of urban consumers, as global brand proliferation is prominent in urban areas of India relative to rural areas (Srivastava, Dey, and Balaji, 2020).

 The current study is not in the context of multi-cultural study, hence the respondents included in this study are only from India. And involving respondents from other countries in this study does not justify the scope of research. However, this could be an avenue for further research. 

**Response to Query no 6:**

**Scenario of biscuit industry in India**

 India’s biscuit market stood at $3.9 billion in 2016, and is projected to grow at a compound annual growth rate (CAGR) of 11.27 per cent, in value terms, between 2017 and 2022, to reach $7.25 billion by 2022. Moreover, augmented disposable incomes, along with changing lifestyles, increasing awareness regarding healthy diets and changes in food consumption patterns, are some of the other factors expected to propel demand for biscuits over the course of the next five years (2023-2027).

 The biscuits and cookies industry in India has been growing at a CAGR of 10 per cent for the last three years, and is currently valued at Rupees 145 billion. India is currently the world’s largest biscuit consuming nation. Compared to other fast-moving consumer goods (FMCG) products, the penetration of biscuits and cookies, in both the urban and rural areas, is quite high 94 percent and 83 percent, respectively (Kachave, 2018; De Mooij, 2021, Junaid et al., 2022).

# Reviewer report: Reviewer response for version 1

**DOI:** 10.5256/f1000research.143913.r169448

**Published:** 2023-05-02

**Authors:** Savita Bhat

**Affiliations:** 1 School of Humanities, Social Sciences and Management, National Institute of Technology Karnataka, Surathkal, Karnataka, India

**Summary of the Article:**

 The study analyzes the effects of consumer dispositional attitudes on the purchase intention of global and local brands. It explains how consumer ethnocentrism and consumer cosmopolitanism form attitudinal dispositions towards brands. The study considers the FMCG product category of biscuits with Parle G as a local brand and Unibic as a global brand. The data from 756 respondents are collected from two metropolitan cities of India, Bengaluru and Chennai, using a questionnaire survey method. The data is analyzed using Partial least squares structural equation modeling (PLS-SEM) approach. The study has performed the reliability assessment and discriminant validity tests using appropriate measures. The results, discussions, conclusion and limitations are also presented.

 Overall, the study is timely, and the results of the study will be helpful for researchers and marketers in an emerging market like India.

 The following are some of the suggestions to improve the study.

1. In Table 9 on Hypothesis outcomes, it is clear that the study does not support the H1a hypothesis. Hence, in the discussions, the authors may avoid stating - In this study, consumer ethnocentrism forms a negative attitude toward global brands (H1a).
2. Further, the path coefficient from CET to ATBL is statistically significant with a negative sign (-0.568); it seems contradictory to the H1b hypothesis. The authors can clarify this.

The conclusion section can be appropriately modified after addressing the above two points.

Is the work clearly and accurately presented and does it cite the current literature?

Yes

If applicable, is the statistical analysis and its interpretation appropriate?

Partly

Are all the source data underlying the results available to ensure full reproducibility?

Yes

Is the study design appropriate and is the work technically sound?

Yes

Are the conclusions drawn adequately supported by the results?

Partly

Are sufficient details of methods and analysis provided to allow replication by others?

Yes

Reviewer Expertise:

Emerging economies; Firm competitiveness; quantitative techniques in economics and management

I confirm that I have read this submission and believe that I have an appropriate level of expertise to confirm that it is of an acceptable scientific standard.

# Response

**Published:** 2023-05-06

**Authors:** Venugopal Shanbhogue

**Affiliations:** Manipal Institute of Management, India

1. Since hypothesis H1a is not supported in this study as shown in table 9, the sentence in the discussion section – “In this study, consumer ethnocentrism forms a negative attitude toward global brands (H1a)” will be removed.

 2. Although the path coefficient of CET=>ATBL is (-0.568) negative for hypothesis H1b as shown in the table 8, the relationship is statistically significant, the hypothesis H1b was formulated based on previous literature which suggested a positive relationship between consumer ethnocentrism (CET) and attitude towards local brand (ATBL). A study by Sharma *et al*. (1994) argues that the higher the product necessity lower is the influence of ethnocentrism on purchase intention among consumers. For example, ethnocentric consumers evaluate products that are a "necessity" differently compared to the products which are owned as status symbols. Since this study included only low involvement product (Biscuit) which is not a status symbol which explains the negative path coefficient between consumer ethnocentrism (CET) and attitude towards brand (ATBL).

**Conclusion**

 The theoretical and managerial implications of this study is significant for marketers especially those working in an emerging market like India as this study has examined the role of consumer disposition on attitude formation and purchase intention. From theoretical perspective, this study developed and tested a conceptual model based on attitudinal disposition and purchase intention. It suggests that ethnocentric consumers have a strong sense of oneness among their in-group, significantly support local culture, traditions, and economy which leads to higher purchase intention towards local brands and also the finding suggests that ethnocentric effect reduces for low involvement products because of increased necessity as seen in this study. It is also noted from the findings that cosmopolitan consumers do not have a negative attitude towards local brands which implies that if there is an alternative to the global brand in the market, cosmopolitan consumers will not out rightly reject the local brand in favour of global brands.

 From managerial contribution, this study can help managers understand the phenomenon of positive attitude formation which in turn leads to purchase intention. The strategies could be developed in accordance with these findings to leverage the target consumer groups. Consequently, these research findings are valuable from more than just an academic standpoint and also for managers/practitioners of brands in an emerging economy like India to strategically plan marketing campaigns, promotions, and positioning of their brands. From managerial implications’ perspective, it can be concluded that managers need to adopt a flexible strategy in order to excite the consumers to buy their products and at the same time make their brand more appealing not only at home but also in foreign markets (Zeugner-Roth *et al*., 2015).
