# Supplementary material for: Effects of Consumer Dispositional Attitude on Purchase Intention in an Emerging Market
Source: F1000Res. 2023 Apr 12;12:384. [Version 1] doi: 10.12688/f1000research.131103.1 (PMC10904944; doi:10.12688/f1000research.131103.1)
Supplement: Peer review history

# Reviewer report: Reviewer response for version 1

**DOI:** 10.5256/f1000research.143913.r179017

**Published:** 2024-06-12

**Authors:** Thamaraiselvan Natarajan

**Affiliations:** 1 Department of Management Studies, National Institute of Technology Tiruchirappalli, Tiruchirappalli, Tamil Nadu, India

The study is a very interesting read. It explores on effects of Consumer Dispositional Attitude on Purchase Intention in an Emerging Market like India. The authors have collected data from respondents of major metropolitan cities Bangalore and Chennai using the convenience sampling method. They have relied upon the SMART PLS software for their analysis.

 Their results give implications for the Marketing managers/practitioners to focus on consumers forming attitude in consumers’ minds so that this attitude formation leads to a purchase decision. The results also imply that attitude formation is the first step in consumers’ behavioral decision making a purchase decision. 

 Overall. given the efforts taken by the authors to write this manuscript involving robust methodology, I feel the manuscript is ready for publication.

Is the work clearly and accurately presented and does it cite the current literature?

Yes

If applicable, is the statistical analysis and its interpretation appropriate?

Yes

Are all the source data underlying the results available to ensure full reproducibility?

Yes

Is the study design appropriate and is the work technically sound?

Yes

Are the conclusions drawn adequately supported by the results?

Yes

Are sufficient details of methods and analysis provided to allow replication by others?

Yes

Reviewer Expertise:

Marketing

I confirm that I have read this submission and believe that I have an appropriate level of expertise to confirm that it is of an acceptable scientific standard.
